# Internet of Things Buttons for Real-Time Notifications in Hospital Operations: Proposal for Hospital Implementation

**DOI:** 10.2196/jmir.9454

**Published:** 2018-08-10

**Authors:** Peter Ray Chai, Haipeng Zhang, Christopher W Baugh, Guruprasad D Jambaulikar, Jonathan C McCabe, Janet M Gorman, Edward W Boyer, Adam Landman

**Affiliations:** ^1^ Division of Medical Toxicology Department of Emergency Medicine Brigham and Women's Hospital Boston, MA United States; ^2^ The Fenway Institute Boston, MA United States; ^3^ Innovation Hub Brigham and Women's Hospital Boston, MA United States; ^4^ Partners Information Systems Sommerville, MA United States

**Keywords:** Internet of Things, operations, hospital systems, health care

## Abstract

**Background:**

Hospital staff frequently performs the same process hundreds to thousands of times a day. Customizable Internet of Things buttons are small, wirelessly-enabled devices that trigger specific actions with the press of an integrated button and have the potential to automate some of these repetitive tasks. In addition, IoT buttons generate logs of triggered events that can be used for future process improvements. Although Internet of Things buttons have seen some success as consumer products, little has been reported on their application in hospital systems.

**Objective:**

We discuss potential hospital applications categorized by the intended user group (patient or hospital staff). In addition, we examine key technological considerations, including network connectivity, security, and button management systems.

**Methods:**

In order to meaningfully deploy Internet of Things buttons in a hospital system, we propose an implementation framework grounded in the Plan-Do-Study-Act method.

**Results:**

We plan to deploy Internet of Things buttons within our hospital system to deliver real-time notifications in public-facing tasks such as restroom cleanliness and critical supply restocking. We expect results from this pilot in the next year.

**Conclusions:**

Overall, Internet of Things buttons have significant promise; future rigorous evaluations are needed to determine the impact of Internet of Things buttons in real-world health care settings.

## Introduction

### Background

Simple repetitive tasks when done manually are often time consuming and can be overlooked or simply forgotten. In a hospital setting, staff perform multiple parallel processes hundreds to thousands of times a day that often require minimal margin of error [1]. Execution of these tasks may be interdependent; without a notification or completed process in an operating cascade, final completion of a task may be delayed or the task may remain incomplete. One possible way to improve hospital staff efficiency and reduce the chance of error with these repetitive tasks is to leverage Internet of Things (IoT) devices. IoT is the “interconnection via the internet of computing devices embedded in everyday objects, enabling them to send and receive data” [2]. An IoT-enabled button can send automatic, reliable, just-in-time notifications or trigger one or more tasks when pressed.

A well-known example of IoT buttons is Amazon’s “Dash” button that enables consumers to quickly reorder specific products through Amazon. Amazon and product manufacturers expect that consumers will place Dash buttons at the location where products are used; when a product’s supply is depleted, a simple press of the Dash button orders a refill. For example, an Amazon Dash button for laundry detergent might be attached to the consumer’s washing machine. When the laundry detergent is running low, the consumer presses the Dash button and laundry detergent is automatically reordered.

IoT buttons can be configured to perform a wide range of actions extending beyond internet product ordering [3,4]. In the hospital, IoT buttons may be a cost-efficient, intuitive, and scalable method to automate repetitive, commonplace hospital tasks and provide real-time insight into daily hospital operations. For example, IoT buttons can be configured to deliver messages to housekeeping, nurse managers, and administrators upon patient discharge, coordinating an efficient bed turnover process while recording each step of the process. These recorded data can then be analyzed to identify further process improvement opportunities.

Little data and almost no protocols exist regarding the real-world usage and operationalization of IoT buttons in hospitals. In this paper, we describe potential applications of IoT buttons to streamline and evaluate hospital operations as well as describe technological considerations, such as data security requirements. We also propose a framework that health care systems can use for IoT button deployment.

### Internet of Things Buttons

**Figure 1:**
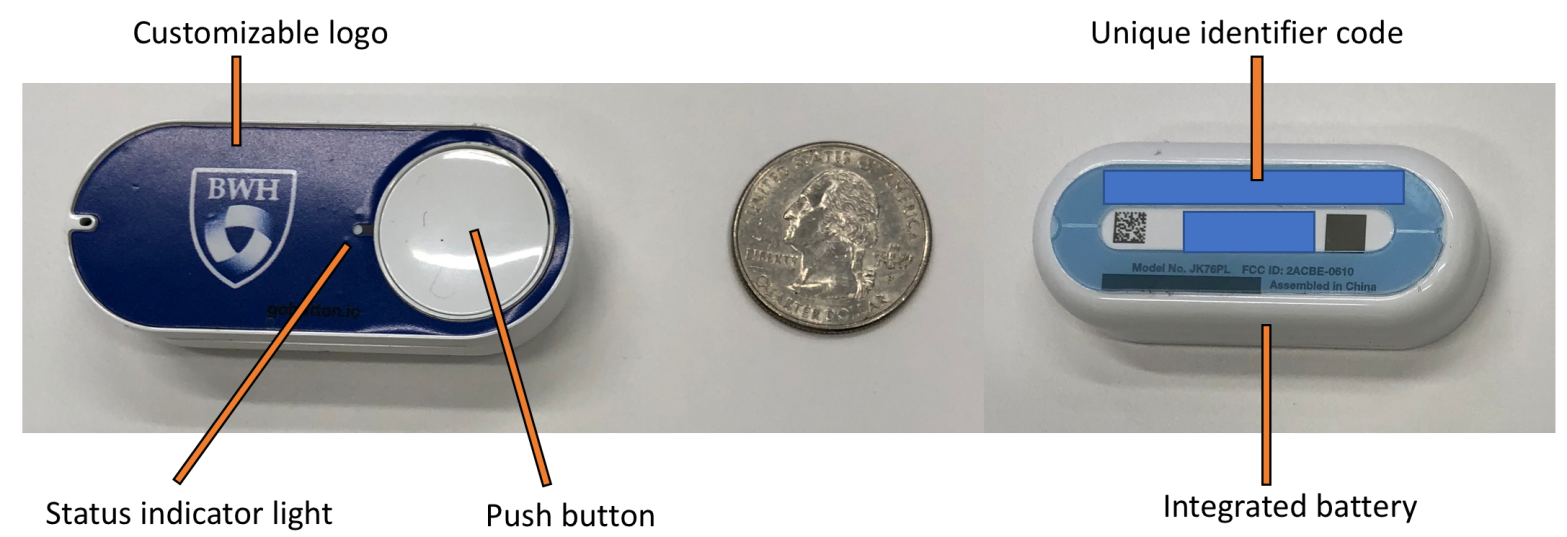
An Internet of Things (IoT) button. A United States quarter is pictured for scale.

IoT buttons are small and unobtrusive (approximately the size of a stick of gum; Figure 1) and are available through a variety of commercial vendors using a range of technologies. IoT buttons can be used for very specific purposes, such as a button located in a patient’s room that, when pressed, indicates that room cleaning is needed. Alternatively, buttons can have more general purposes, such as a patient call button that summons assistance, but does not specify the precise reason for the assistance.

**Figure 2:**
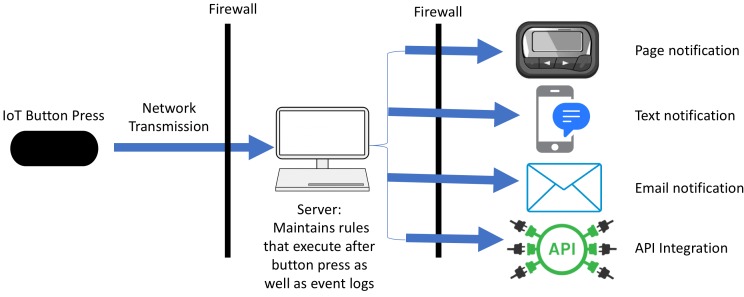
Schematic of the process flow of an Internet of Things (IoT) button press. API: application programming interface.

Pressing the IoT button sends a preprogrammed message through a network (often wireless) to a server that can send customizable notifications. Notifications might generate a standard short message service text message, an email, or a page (Figure 2). Additionally, notifications can be extended almost infinitely by calling application programming interfaces (APIs), software methods that allow computer systems to exchange information.

A single IoT button may also have the ability to perform multiple, distinct actions through different types of button presses (single button press, double button press, and long press). For example, a single press of the button may send a notification via email, a double press can send a different message to a pager, whereas a long press may log an event into the electronic medical record (EMR) through an API. Using the patient discharge and room cleaning application discussed earlier, a single button press may notify housekeeping that the room needs to be cleaned, whereas a double press may record when housekeeping has completed the task. Finally, a long press can call the EMR’s API to update the room’s status.

The IoT buttons can also provide feedback to users in real time. One IoT button vendor embeds a multicolor indicator light on the device. The light flashes white to indicate that a button press was detected and then changes to green to signal successful delivery of the notification. If a notification is not delivered, the light flashes red to alert the user to an error. In order to prevent inadvertent or deliberate repetitive presses, buttons should have the ability to lock out after a defined number of presses or period of time.

Button presses can be logged in a database and subsequently used for data visualization and analytics. The database could record which button was pressed, type of press, location of the button, and date or time of action. Off-the-shelf analytics tools can facilitate data summarization and visualization. Further, the data could be used for more complex analytics. Returning to the earlier room cleaning example, the stored data could be analyzed to determine the average room cleaning time by comparing the time of the initial room cleaning request (single button press) with the time of the housekeeper response (double button press). These analyses may in turn help influence future staffing decisions, such as the number of staff, location of work, and task schedules.

### Existing Hospital Notification Systems

Many different notification systems currently exist in health care systems (Table 1). These notification systems may be devices like a patient call button, quick response code, or technological measures like a hospital paging portal. The widespread conversion of hospital medical records into EMRs has also fueled the development of EMR rules and dashboards to manage EMR-based notifications. IoT buttons are a highly adaptable notification system that can potentially supplement or replace other existing hospital notification systems. Buttons can be placed in various environments and programmed to deliver custom messages in response to specific tasks. Although the IoT button is a physical device, activating the button can trigger a cascade of tasks in other systems like the medical record or a Web-based paging portal. Additionally, IoT buttons can measure their own usage through usage logs.

**Table 1:** Advantages and challenges of existing hospital notification systems and Internet of Things (IoT) buttons.

Notification system	Current application	Advantages	Challenges
QR code^a^ readers	Notification systems	Universal code that can be accessed through mobile phones or dedicated barcode scanners	Multistep process to activate Requires a mobile phone with QR reading capability or barcode scanner
Patient call button	Patient-facing notification system to call nursing staff	Recognizable device with simple user interface	No context for notifications
EMR^b^-based notification rules	Signaling completed tasks based on EMR changes	Improved process flows via EMR events Can be applied quickly through a hospital	Task must be based on EMR change Each new application requires programming
Web-based paging system	Sending custom notifications to providers, hospital staff	Web portal allows for access anywhere	Requires accessing paging system to deliver each notification
IoT Buttons	Patient or staff facing	Notification delivered with push of a button Notifications can be simple or complex actions	Requires installation of buttons Security and privacy issues Programming or configuration of buttons required

^a^QR code: quick response code.

^b^EMR: electronic medical record.

## Methods

### Potential Applications in Hospital Operations

Multiple potential applications for IoT buttons exist in hospital operations. We classified applications into two categories: patient and hospital staff applications, based on the primary intended user group (Textbox 1).

For patients, an IoT button may be a potential replacement for the traditional patient call button. A small, mobile wireless IoT button, or even an IoT button affixed to a rail of a hospital bed, that is given to a patient can be programmed to call for help. While a traditional hospital call button conveys a single piece of information (ie, the patient needs assistance), an IoT button can communicate a greater breadth of information. For example, a patient may use a single button press to call for help, but a responding nurse could then provide a long press to indicate that the call has been addressed or provide two presses to request additional help. The ease of button use may improve the reliability and validity of patient reported outcomes [5,6].

Hospital staff applications of IoT buttons can help focus not only on streamlining notifications but also on gathering real-time data that can be used for workflow quality improvement. For example, an IoT button on a linen cart can help hospital staff notify housekeeping when linens need to be restocked. Button presses for restock requests can be aggregated and analyzed to determine the patterns of usage. These data could be used to recommend and justify staffing changes to fulfill supply requests and subsequently to evaluate the response to such staffing changes. Similarly, IoT buttons can be used to analyze key operational chokepoints in patient flow. Button presses may flag inpatients who are ready for discharge on morning rounds, sending notifications to key individuals like case management, social work, and housekeeping that facilitate discharge and bed turnover. Similarly, IoT buttons may be leveraged to flag patients in the emergency department who are ready to be admitted, delivering notifications to responding clinicians, bed control specialists, and transport, thereby initiating multiple cascades of tasks required to admit a patient to the hospital.

**Potential applications of Internet of Things buttons to improve hospital operations classified by primary user group.**

**Patient applications**

- Restroom cleaning alerts
- Use as a call button to:
  - Contact clinical teams
  - Report distress
  - Contact other hospital staff including research teams

**Hospital staff applications**

- Supply chain restock
- Notifications of critical orders or events, such as flagging patients for discharge
- Notifications to specific hospital services, such as respiratory, phlebotomy, and information technology support
- Optimizing hospital bed turnover
- Identifying potential research participants in the hospital
- Initiating a bed request for hospital admission

## Results

We are planning several pilots to evaluate the use of IoT buttons in a hospital system and to help further identify best practices for deploying IoT buttons. Currently, we are piloting IoT buttons in hospital public restrooms to assess and improve their cleanliness. We are planning to use IoT buttons to understand demand and patterns of restocking for patient equipment like stretchers and wheelchairs in the emergency department. These interventions will also allow for real-time assessment of response time from staff. We anticipate initial results in the next year regarding the feasibility and acceptability of these pilots.

## Discussion

### Technological Considerations

#### Connectivity

Ensuring secure and reliable network connectivity is an important consideration in IoT button deployments. Unlike streaming applications, IoT buttons do not require continuous connectivity as they only need to transmit data upon button press. By connecting IoT buttons to the network on demand, battery life can be conserved. One vendor uses this strategy to achieve a battery lifetime of 2 years or 2000 clicks [7].

Deploying a large number of IoT buttons in a hospital environment has the potential to overwhelm network capacity if the proper engineering expertise is not consulted. Therefore, an initial, limited deployment should be considered to test the feasibility of IoT button use as well as the network bandwidth requirements. Additionally, the use of dedicated networks for IoT buttons may help ensure efficient IoT button performance and also minimize the chance of unintended consequences, such as network disruptions, to the primary hospital network.

#### Button Management Systems

Some vendors offer button management systems (BMS), Web-based administrator interfaces, or consoles for IoT button programming and monitoring. From a hospital perspective, important administrator features include audit trails, battery life monitoring, and the ability to group buttons by use case. Audit trails or log data are particularly important for quality assurance, whereas early warning systems, which alert an administrator to low battery life, ensure button functionality and availability. Similar to how website content management systems enable individuals to control content in specific website sections, the BMS should include controlled access to groups of buttons by characteristics such as department, physical location, or use case. BMS should also be flexible and intuitive so that users with limited technical proficiency can modify button configuration, whereas more sophisticated users can customize button functionality using programming languages. In addition, BMS support for batch programming will facilitate rapid deployment of a large number of buttons with identical functionality.

#### Privacy and Security

When considering the use of IoT buttons, hospital systems should understand the potential privacy and security risks. Privacy breaches can occur when unencrypted button messages containing protected health information (PHI) are intercepted. Network security can be compromised when IoT buttons are used as an entry point into hospital networks or used as a distributed denial of service (DDoS) attack. Understanding these risks and creating strategies to effectively mitigate them are central to safely deploying IoT buttons (Table 2).

Patient privacy may be compromised if button notifications contain PHI. For example, if an IoT button is configured to send the following message “John Smith in Room 300 needs help,” interception of these data could reveal not only the presence of a specific patient within a hospital but also pinpoint the patient’s location. To protect against breaches in patient privacy, messages should be carefully constructed to avoid PHI. If PHI or other sensitive information needs to be transmitted, buttons should utilize modern encryption protocols.

Open firewall ports, used to deliver button notifications, may provide a portal to enter a hospital network and steal critical health information or conduct malicious attacks against hospital infrastructure. To minimize the chance of these attacks, IoT buttons can be programmed to only connect briefly to a hospital network, while a notification is being sent, minimizing the time a critical firewall port is open. Hospitals may also consider isolating IoT buttons on an independent network to protect hospital infrastructure from infiltration.

In order to transmit sensitive notifications containing PHI, IoT buttons should securely connect to an encrypted wireless network. Many IoT buttons support the Wi-Fi-Protected Access 2 (WPA2) mechanism, which requires a single, preshared password to connect to the wireless network. While the WPA2 mechanism conveys some protection, a hacker who learns a single preshared key can compromise the entire system, leading to reprogramming of IoT button functions, or disabling an IoT button network. IoT buttons should also support Wi-Fi-Protected Access-enterprise (WPA-enterprise) encryption system that requires a user to enter a unique username and password to log into the network, providing an additional layer of security necessary in networks that transmit confidential information. This way, even if the hackers learn the password of one IoT button, they cannot compromise the entire system. Another option that can reduce the chance of malicious activities is using IoT buttons with an integrated cellular network, bypassing the need to connect the IoT button to an institution’s corporate network.

Malicious users could also conduct DDoS attacks by sending rapid, high-volume button presses from one or more IoT buttons in an attempt to overwhelm the hospital network [8]. As noted previously, buttons can be programmed to lock out for a period of time after each button press, which can help prevent DDoS attacks. In addition, button availability can be limited to appropriate users. For instance, buttons that communicate sensitive information should only be accessible to authorized hospital staff.

**Table 2:** Privacy and security considerations for Internet of Things (IoT) buttons.

Potential concerns	Potential solutions
Privacy and data breach	Communicate deidentified data Use encryption Disallow continuous network connection and data transfer Enable IoT buttons to communicate via cellular networks to avoid integration with hospital networks
Theft	Strategic placement Secure installation
Distributed denial of service (DDoS) attacks	Lockout times on buttons to prevent DDoS based on number of button presses IoT buttons placed on separate network
Failure	Staggered adoption with careful testing of failure rates Initial use in conjunction with existing notification methods

**Figure 3:**
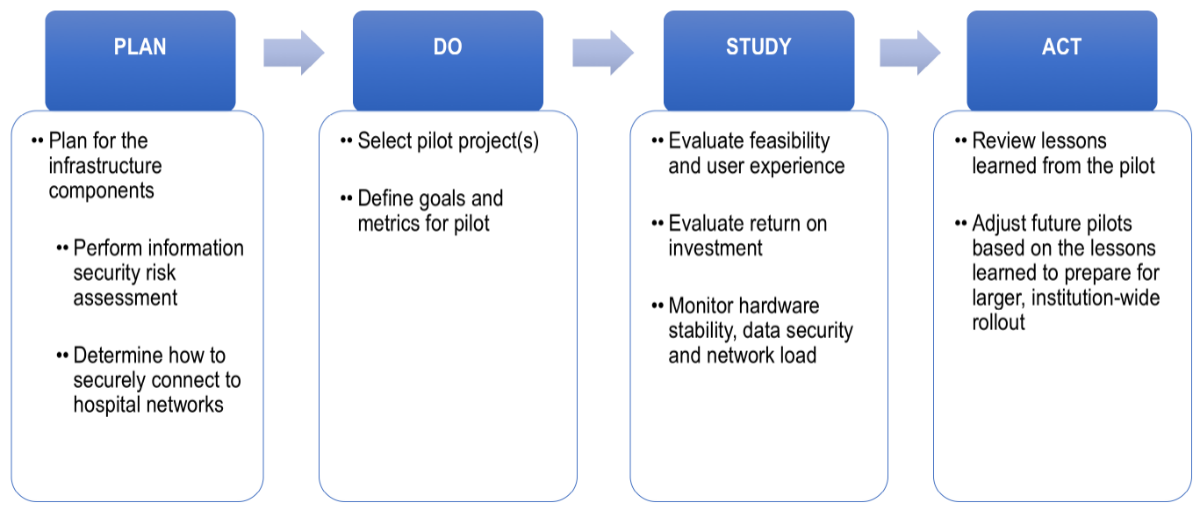
A proposed framework to deploy and evaluate the impact of Internet of Things (IoT) buttons in a hospital.

### A Framework for Internet of Things Button Deployment

For health care organizations that seek to deploy IoT buttons across a large hospital, we recommend the following steps grounded in the Plan-Do-Study-Act (PDSA) method (Figure 3) [9,10]. PDSA is often used to accelerate quality improvement initiatives to rapidly test changes by planning them, implementing them, observing the results of the intervention, and iterating the changes based on what is learned [11]:

- Plan: The first step in an IoT button implementation is to plan for the infrastructure components. Since the buttons will require network connectivity, hospital information security officers should be engaged to perform a risk assessment of the technology and mitigate any high priority risks identified.
- Do: The team should select a limited, yet important, task that would benefit from a brief IoT button pilot.
- Study: The pilot should be evaluated to assess feasibility, user experience with and usage of the IoT buttons, and return on investment. In addition, hardware stability, data security and quality, and network load should be monitored during the pilot.
- Act: Lessons learned from the pilot, including technical, workflow, and other components of the sociotechnical model for health information technology, should be carefully reviewed [12]. To prepare for larger roll-outs, IoT button processes and protocols should be adjusted based on these lessons learned.

### Limitations of Internet of Things Button Deployment

While there are many exciting applications for IoT buttons in the hospital setting, limitations also exist. First, not all hospital tasks are well suited for an IoT button intervention. Further, rapid, widespread deployments may lead to “button fatigue” as users are confronted with a bewildering array of buttons [13,14]. Therefore, a dedicated governance process, including the project team, information technology staff, and institutional leaders, is essential to triage new button requests for appropriateness and to help reduce risk of button fatigue. Second, it is important to understand button reliability and impact on technical infrastructure. The reliability of IoT buttons must be understood to ensure that buttons can be safely used for desired tasks. For example, a patient call button must be highly reliable (and may even require US Food and Drug Administration review). Plans must also be developed to ensure adequate network bandwidth to support the desired number of buttons. Third, the presence of “false presses” where a user inadvertently presses an IoT button may still overwhelm an IoT button system. Although timed lockout periods may mitigate the transmission of false presses, refinement of a protocol to detect and manage false IoT button presses is still needed. Fourth, the ethics and privacy implications of IoT buttons remain to be explored. Depending on the method in which IoT buttons are deployed, employers may be able to discover and better understand performance metrics of specific employees (for example, knowing how fast a nurse responds to a patient’s IoT button press when used as a call button). Finally, IoT buttons are physical devices that may be lost, damaged, or stolen; however, these shortcomings may be minimized by changing how the buttons are secured or where the buttons are located or using completely software-based buttons [15].

### Conclusion

IoT buttons may be a valuable tool to help optimize hospital operations and communication for a variety of use cases for both patients and staff. Key technical considerations for a successful deployment include ensuring appropriate network connectivity, selecting a product with a robust button management system, and carefully considering configuration to minimize privacy and security risks. The PDSA framework may guide hospitals starting with a small pilot, iteratively refining the process and, eventually, scaling to the entire organization. IoT buttons have significant promise, outweighing minor limitations, but need to be tested in real-world health care environments and rigorously evaluated to determine their impact.

## Abbreviations

API: application programming interface
BMS: button management systems
DDoS: distributed denial of service
EMR: electronic medical record
IoT: Internet of Things
PDSA: Plan-Do-Study-Act
PHI: protected health information
QR code: quick response code
WPA2: Wi-Fi-Protected Access 2
